# One-Step Green Synthesis of Gold Nanoparticles Using Black Cardamom and Effect of pH on Its Synthesis

**DOI:** 10.1186/s11671-015-1055-4

**Published:** 2015-09-04

**Authors:** Ashwani Kumar Singh, O. N. Srivastava

**Affiliations:** DST Unit on Nanoscience and Technology, Department of Physics, Banaras Hindu University, Varanasi, 221005 India

**Keywords:** Green synthesis, Gold nanoparticles, Natural precursor, pH effect

## Abstract

In the present article, an effective, one-step, and environmentally benign protocol for the synthesis of gold nanoparticles has been discussed. The black cardamom extract is used as a reducing agent for HAuCl_4_.3H_2_O. In order to synthesize gold nanoparticles, an aqueous solution of HAuCl_4_.3H_2_O was mixed with an optimized concentration of black cardamom extract where 1,8-cineole is the dominant component. Choosing black cardamom extract as a reducing agent can be justified under the light of the fact that it has a very fast reducing ability. Gold nanoparticles with different shapes and sizes were synthesized by varying the ratio of AuCl_4_ ions to black cardamom extract. Kinetics of reactions has been evaluated through monitoring of surface plasmon behavior of gold nanoparticles as a function of time. Based on Fourier transform infrared spectroscopy (FTIR) studies, a tentative mechanism of reduction of Au nanoparticles has also been proposed which includes oxidation of 1,8-cineole to 2-oxo-1,8-cineole. Further, a comprehensive study to investigate the effect of pH on the synthesis of Au nanoparticles has been carried out.

## Background

Attaining of unique properties by tailoring the materials at atomic level can be achieved by the process of nanotechnology [1]. There have been impressive developments in the field of nanotechnology in the recent past years, with numerous methodologies formulated to synthesize nanoparticles of particular size and also of shape depending on specific requirement.

Recently, a resurgence of interest in metal nanoparticles has resulted due to their potential applications in the emerging field of plasmonics [2–4]. Plasmonics uses the unique optical properties of metallic nanomaterials to manipulate the transfer of light on the nanoscale and is a promising technology for integrating the large data-carrying capacity of optical interconnects with nanoscale electronic devices.

Metal nanoparticles are known to display tremendous potential for biological and chemical sensing [5–10] and cancer therapy [11]. They can serve as a model system to experimentally probe the effects of quantum confinement on electronic, magnetic, and other fated properties [12–14]. They have also been widely exploited for use in photography [15], catalysis [16, 17], photonics [18], optoelectronics [19], information storage [20], surface-enhanced Raman scattering (SERS) [21–23], and formulation of magnetic ferrofluids [24].

Among various methods available for the synthesis of metal nanoparticles, laser evaporation and chemical reduction are the major ones [25]. However, each method has certain limitations. For example, the use of costly and sophisticated instruments and the problems associated with their handling in case of laser evaporation and also the yields of nanoparticles are quite low. Similarly, chemical reduction method may end up with the adsorption of some toxic chemical species on the surface even though gold nanoparticles are considered biocompatible which needs an additional step of removal of these toxic species. This may have some adverse effects in medical applications. Therefore, there is an urgent need to develop an environmentally benign nanoparticle synthesis protocol. This tempts the researchers in the field of nanoparticle synthesis and assembly to utilize some eco-compatible natural compounds for the reduction of Ag- and Au-containing salts for the synthesis of Ag and Au nanoparticles [26, 27]. Recently, microorganisms mediated nanoparticle synthesis and gained much importance because of biocompatibility and facile assembly of nanoparticles [28–33]. Sastri et al. opened an avenue to the synthesis of metal nanoparticles by eukaryotic organisms [34, 35]. Later, they carried out extracellular synthesis of Ag and Au nanoparticles, using fungi [36–38]. They demonstrated that the shift from bacteria to fungi as a means of developing ‘natural nanofactories’ has the added advantage for the processing and handling of the biomass.

Further, plant extracts have received considerable attention as an effective reducing agent for Ag- and Au-containing salts to synthesize the Ag and Au nanoparticles. Jose-Yacaman et al. have reported the first living plant-mediated synthesis of silver and gold nanoparticles [39]. Similar biosynthesis of nanoparticles was achieved by Sastri et al. by using plant leaf extracts, and they explored further potential applications [40]. They studied bio reduction of silver ions and chloroaurate ions by the broth of geranium leaf [41] or neem leaf [42]. They also demonstrated the synthesis of gold nanotriangles from tamarind leaf extract and studied their potential application in vapor sensing [43]. Recently, some scientist synthesized the gold nanotriangles and silver nanoparticles, using aloe vera plant extract [44].

With this literature background, we herein report a novel, eco-compatible, and green synthesis of gold nanoparticles from Au^III^ salts by using extract of black cardamom as a natural reducing agent. Black cardamom is widely used extensively in India, in foods, beverages, mouth fresheners, and native medicine. Black cardamom has been used as reducing agent in our synthesis protocols. Synthesis of gold nanoparticles by employing Black cardamom as a reducing agent and the effect of pH on synthesis have been discussed and described in this article.

## Experimental Details

### Materials and Methods

HAuCl_4_.3H_2_O, procured from Sigma Aldrich and dried black cardamom easily available in commercial market, have been adopted as staring materials. These materials have been used without any further purification.

### Preparation of Black Cardamom Extract

In the synthesis protocol adopted here, black cardamom has been used as a reducing agent, prepared by simply dipping 5 gm of it for 24 h into 100 mL of double-distilled water. The solid content was filtered out, leaving the residual extract of dark brownish color. This extract was further used in consecutive steps for the synthesis of Au nanoparticles. We have further studied the following aspects of synthesis in some details:

1. The effect of varying amount of reducing agent
2. The role of pH on the synthesis of Au nanoparticles

For the first investigation, the synthesis steps involve the mixing of a 50-mL (0.001 M) aqueous solution of HAuCl_4_.3H_2_O to different amounts of black cardamom extract under constant stirring of 250 rpm at room temp. Three different concentration ratio, 1:1, 1:0.5, and 1:0.1 have been selected for this purpose. Subsequent changes in color (which occurs within minutes) indicate the successful synthesis of Au nanoparticles.

### Synthesis of Gold Nanoparticles

To study the effect of varying the amount of black cardamom extract on the synthesis of nanoparticles, three samples with different ratios of HAuCl_4_ and black cardamom extract (1:1, 1:0.5, 1:0.1) have been prepared. Subsequent changes in color within minutes, depending on the shape and size of the particles, clearly indicate the formation of gold nanoparticles. Solutions were further centrifuged at 5000 rpm for nearly 10 min. Gold nanoparticles thus obtained were collected and resuspended in double-distilled water, and in order to remove impurities, this process was repeated for three times.

### XRD Study

To investigate the phase formation and crystal structure, X-ray diffraction (XRD) analysis has been carried out by using X-ray diffractometer (PAN—analyst BV, the Netherlands with a built in graphite monochromator) using Cu Kα radiation with Ni filter in a wide range of Bragg angle (20 < 2θ < 80). For this purpose, the Au nanoparticles obtained through the process described above were placed on a glass disk (~5 mm diameter), allowed to dry, and then mounted in the specimen port of diffractometer.

### TEM Analysis of Gold Nanoparticles

After the completion of a successful synthesis process, suspended centrifuged particles have been sampled for TEM analysis. In this process, the samples of gold nanoparticles were prepared by placing a drop of obtained suspension after centrifugation on the Formvar-coated copper grids. The grids were further dried and used for TEM analysis. For shape, size, and microstructural details of these as-synthesized gold nanoparticles, TECNAI 20 G^2^ electron microscope, operated at an accelerating voltage of 200 kV, has been used.

### UV-Visible Spectroscopic Studies

The evolution of nanoparticles from AuCl_4_^−^ ions has been observed through monitoring the UV-visible spectra of synthesized Au nanoparticles. The samples were analyzed by employing Perkin Elmer Lambda 750S UV-Visible spectrometer with a resolution of 1 nm.

## FTIR Spectroscopic Study

Fourier transform infrared spectroscopy (FTIR) spectra of black cardamom extract, before and after bio reduction of Au nanoparticles, have been taken by employing Perkin Elmer Spectrum 100 instrument for unrevealing the mechanism of formation of Au nanoparticles through the reduction of AuCl_4_^−^ ions.

## Results and Discussions

### XRD Analysis

**Fig. 1:**
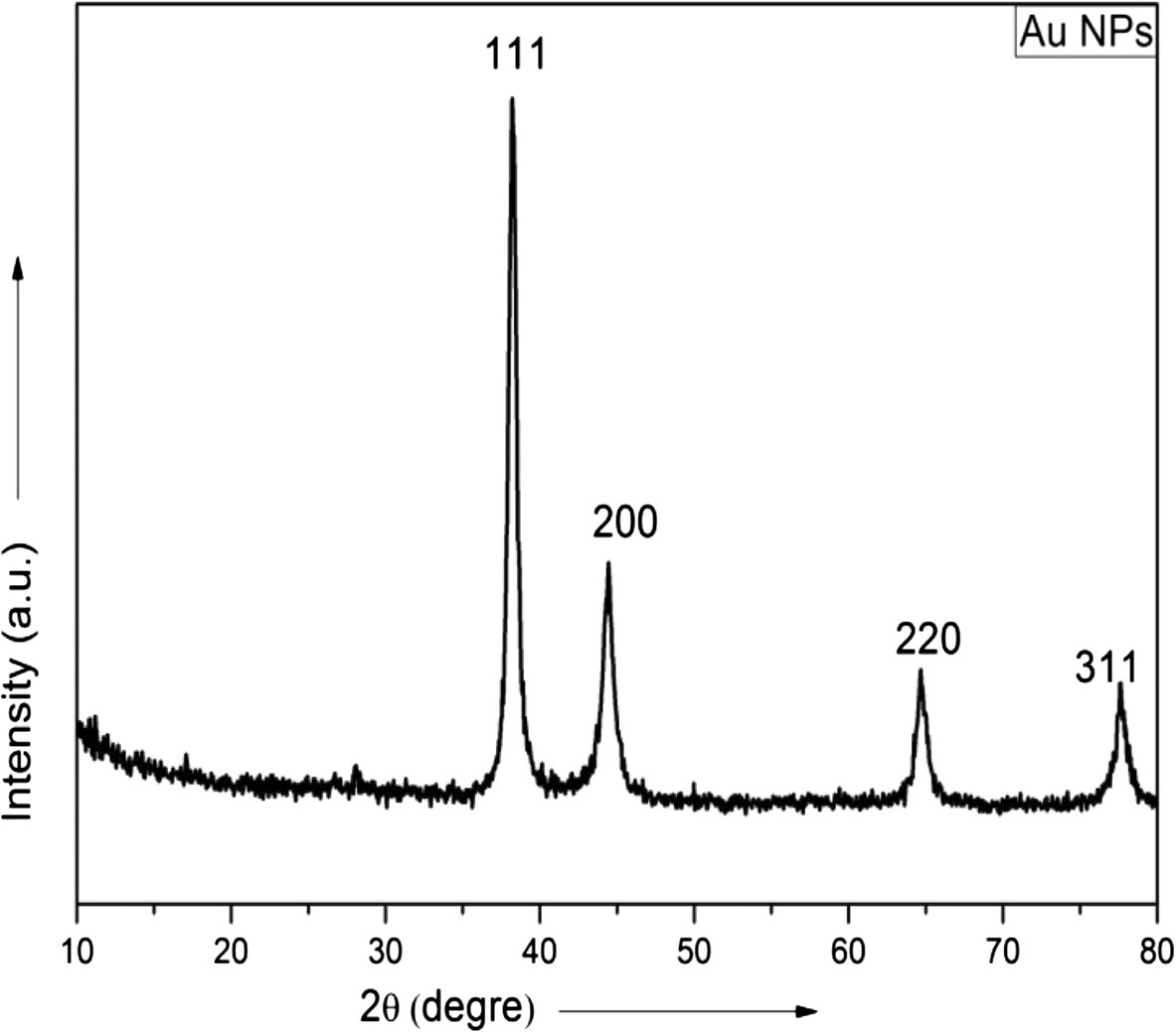
XRD pattern of as-synthesized gold nanoparticles

Figure 1 shows the XRD pattern of as-synthesized Au nanoparticles. It shows intense peaks at 38.49°, 42.21°, 63.88°, and 78.34°. Effects to index, these lines showed that they were explicable only based on Au lattice structure. Thus, the as-synthesized material is nanoparticles of Au.

### UV-Visible Spectroscopy of Synthesized Gold Nanoparticles

Instant change in color of aqueous solution of HAuCl_4_.3H_2_O after the addition of black cardamom extract clearly indicates the formation of gold nanoparticles. One milliliter of this reaction mixture, diluted with 3.0 mL of double-distilled water, has instantaneously been taken for the UV-visible spectroscopic investigations. Three different samples of gold nanoparticles have been prepared with varying ratio of HAuCl_4_.3H_2_O to black cardamom extract (i.e., 1:1, 1:0.2, and 1: 0.1) resulting in distinct colors arising due to the difference in shape and size of nanoparticles

**Fig. 2:**
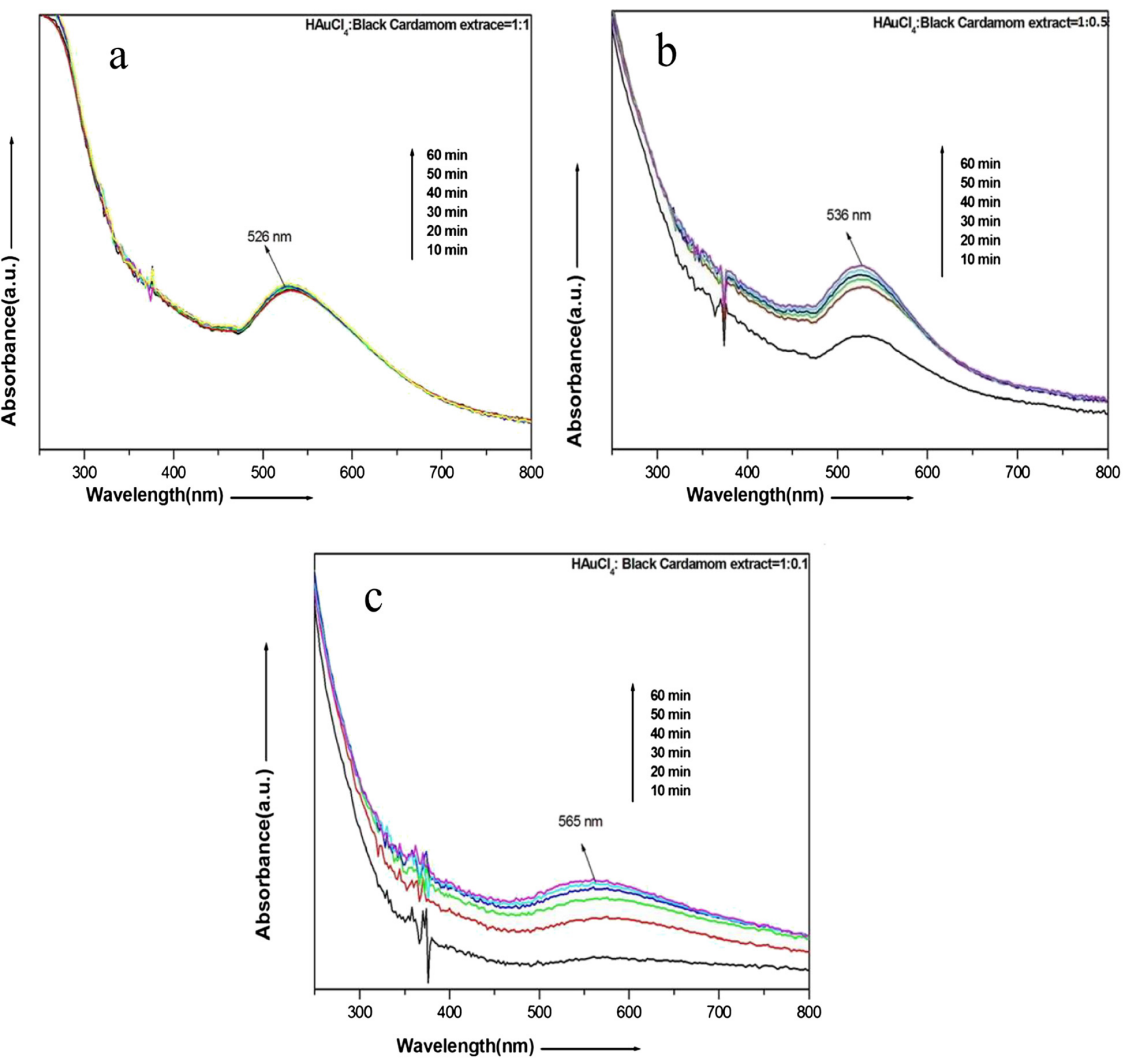
**a**–**c** UV-visible spectrograph of gold nanoparticles synthesized in different ratio of HAuCl_4_ and black cardamom extract

To investigate the evolution of nanoparticles with time in the content ratio of HAuCl_4_ to black cardamom extract (1:1), a series of spectra have been recorded at the interval of every 10 min as shown in Fig. 2. Figure 2a shows the UV-visible spectra of gold nanoparticle, synthesized with HAuCl_4_ to black cardamom extract ratio of 1:1. Here, all the recorded spectra of gold nanoparticles at the interval of 10 min overlap with each other, which indicates the fast formation of gold nanoparticles as the reaction (formation of nanoparticles) gets completed fully within first 10 min. A sharp peak referred as absorption maxima appeared at around 526 nm due to result of interaction of electromagnetic radiations with surface plasmons of gold nanoparticles. Figure 2b, which is the absorption spectra of gold nanoparticles, synthesized with AuCl_4_ to black cardamom extract ratio of 1:0.5, clearly reveal that the absorption maxima has been shifted to 536 nm. This red shift (towards higher wavelength) in absorption maxima indicates the reduction in the size of the particles in comparison to the sample 1 (i.e., HAuCl_4_ to black cardamom extract ratio of 1:1). One interesting feature associated within this spectrum is that there is a large gap between the absorbance spectrum recorded at an interval of 10 and 20 min, but it has been seen that after 20 min, there is no significant change in total absorbance and all other spectra coincide.

This leads to the conclusion that reaction took place within first 10 to 20 min. After that, the rate of the reaction gets slowed down with no significant change in total absorbance of reaction mixture. A similar conclusion which can be drawn from Fig. 2c is the absorption spectra of gold nanoparticles, synthesized with HAuCl_4_ to black cardamom extract ratio of 1:0.1. In this case, a broad peak at 565 nm appears. This peak is further red shifted, and initial four spectra are more distinctly separated with respect to the spectra of Fig. 2a, b. Therefore, it can be concluded that the reaction has further been slowed down, and the reaction took nearly 30 to 40 min to get completed. All these spectra shown in Fig. 2 provide an indication to follow a regular pattern with the size of the particles, which allowed us to conclude that as the amount of black cardamom extract in the reaction (i.e., 10 to 1 mL) have been reduced, the size of the particles increased. Another distinct feature of these UV-vis spectra of Au nanoparticles is that it becomes broader around the prime peak as we decrease the concentration of black cardamom extract. This broadening occurs because as we decrease the concentration of black cardamom extract, particles with different shapes and sized begin to form. We know that different sizes of nanoparticles contribute to different positions of SPR maxima. Since UV-vis spectra depict the collective oscillation of surface plasmons of all nanoparticles, therefore, broadening occurs as a result of polydispersity. It has been confirmed in the TEM investigation of synthesized gold nanoparticles.

### TEM Analysis of Gold Nanoparticles

**Fig. 3:**
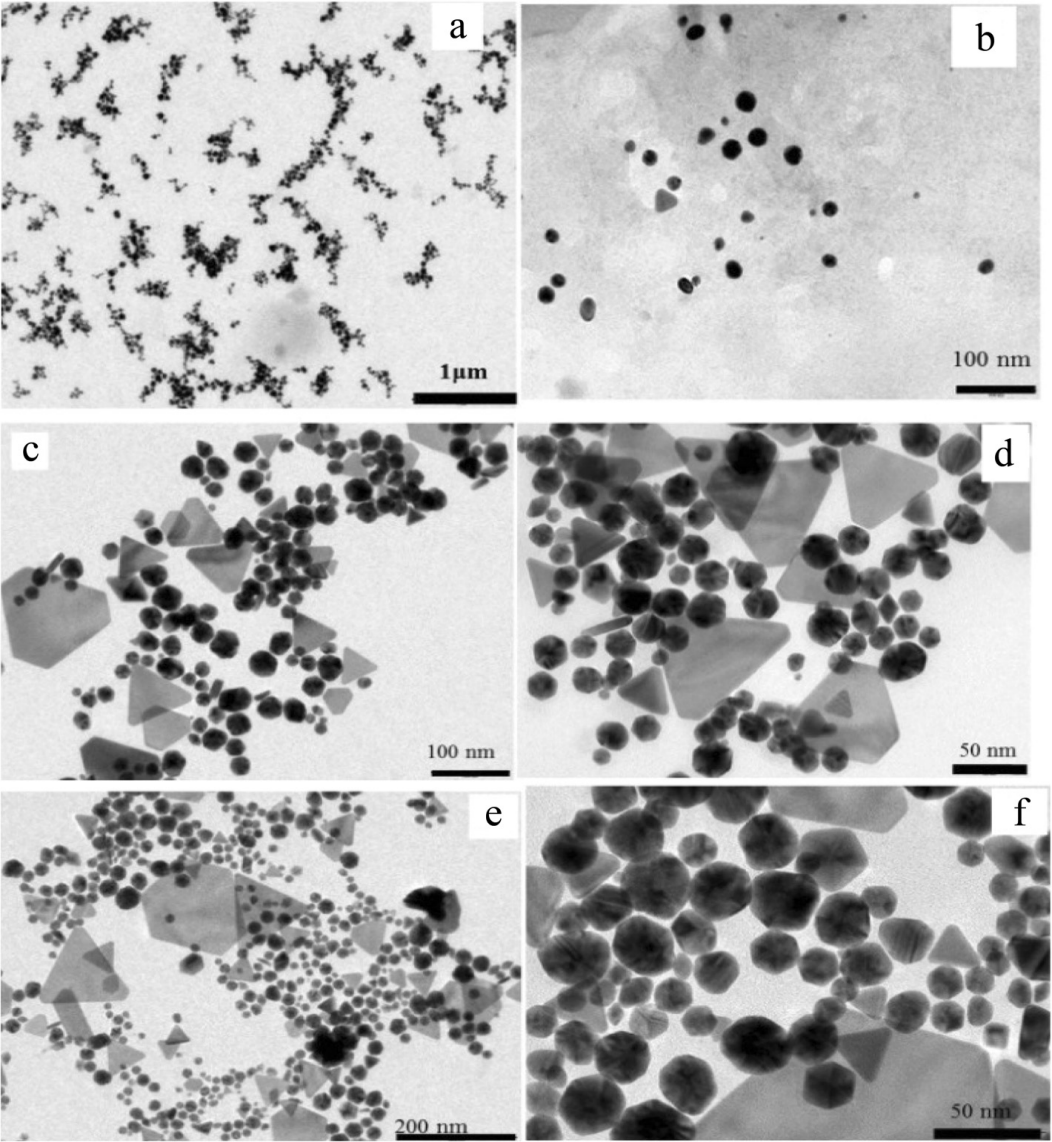
**a**–**f** Transmission electron micrograph of synthesized gold nanoparticles

After a careful investigation of spectra in Fig. 2, prime absorption peak appears to be red shifted with decreasing the concentration of cardamom extract. Besides the prime peak, some small peaks also appear around 375 nm along with the main peaks at 536 and 565 nm. In order to understand these anomalies, intensive TEM analysis of all the three samples has been carried out. Figure 3 depicts the typical transmission electron micrograph of gold nanoparticles synthesized by using black cardamom extract as a reducing agent in different ratios to HAuCl_4_. Figure 3a represents the particles resulting from the reduction of HAuCl_4_ with black cardamom extract in a ratio of 1:1. It has been shown that the particles are lying in reasonably good dispersion. Figure 3b represents a magnified image of these gold nanoparticles at the scale bar of 50 nm, which clearly reveal that the particles are nearly spherical in shape. Sizes of the particles have also been calculated and found to be in the range of 15–20 nm. However, few anisotropic nanoparticles have also been observed but their number in one snap shot are nearly negligible (<2 %). This analysis provides a stand to conclude that the particles, synthesized with HAuCl_4_ and black cardamom extract in the ratio of 1:1, have found to be nearly monodisperse in nature and lying in the size range of 15–20 nm. Figure 3c, d also reveals the formation of gold nanoparticles in the case when HAuCl_4_ and black cardamom extract have been mixed in the ratio of 1:0.5. Two different types of particles are clearly visible in these representative micrographs. One is nearly circular or hexagonal with an average particles size of 20–30 nm and other having some equilateral triangle shapes in small numbers with an average edge length of 70–100 nm.

**Fig. 4:**
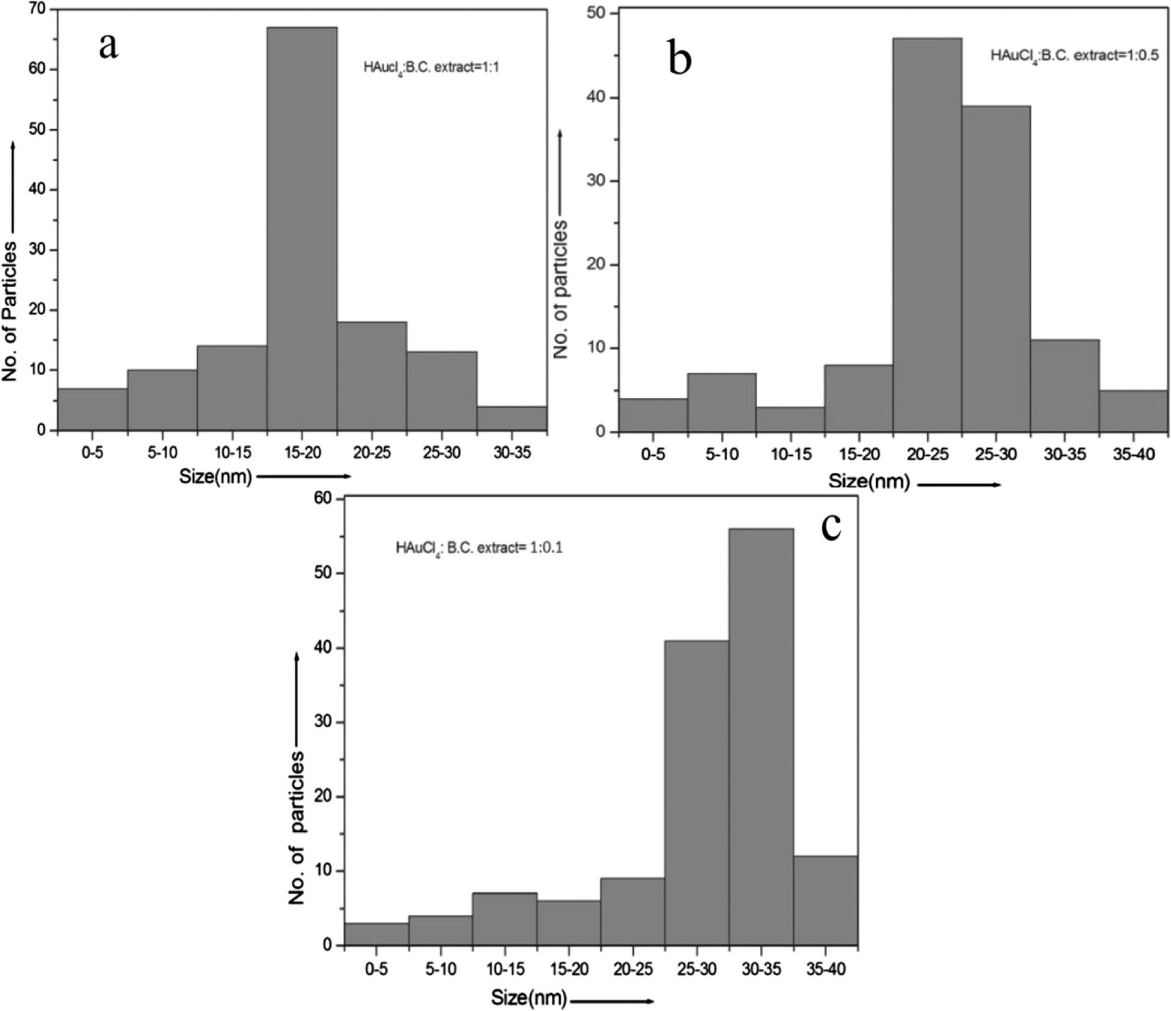
**a**–**c** Histogram plots of gold nanoparticles showing the particle size distribution

From the close observation in Fig. 3a, b to c, d, it can be concluded that the particles in Fig. 3c, d are less monodispersed in comparison to those in Fig. 3a, b. Furthermore, 10 mL of HAuCl_4_ has been mixed with 1 mL black cardamom extract (1:0.1), resulting in the formation of gold nanoparticles shown in Fig. 3e, f. Figure 3e, f represents that the particles have been formed with mainly two shapes, i.e., triangular and spherical. The triangular shaped particles are less in numbers. The probable reason to appear extra peaks at 375 nm in absorption spectra along with main peak is due to the presence of these minority multishaped nanoparticles. Also, the size of the particles increased as amount of black cardamom extract decreased, and it has been found to be in range of 25–35 nm. A detailed study of particular size analysis has been performed using software Image J, as represented through Fig. 4. It distinctly illustrates the distributions of the gold nanoparticles synthesized with different ratios of HAuCl_4_ to black cardamom extract. Figure 4a–c represents the particular size distributions of gold nanoparticles synthesized in the ratios of HAuCl_4_ to black cardamom extract, 1:1, 1:0.5, and 1:0.1, respectively. These histograms explicitly show a gradual increment in the size of synthesized nanoparticles, if the amount of black cardamom extract is reduced during synthesis. These results are in coherence with the results that have found in UV-visible spectroscopic study of these gold nanoparticles synthesized with three different ratios of the HAuCl_4_ and black cardamom extract.

### FTIR Analysis of Black Cardamom Extract

There are few reports available on pharmaceutical composition of black cardamom which indicate that out of 40 identified compounds of black cardamom, 1,8-cineole (65 %), β-Pinene (0.85 %), and α-Terpineol (7.92 %) are the main constituents [45].

**Fig. 5:**
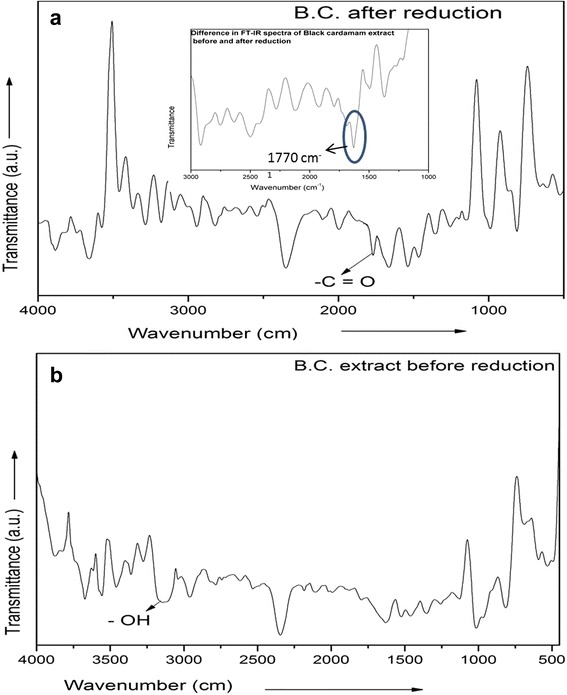
FTIR spectrum of Black Cardamom extract **a** after reduction and **b** before reduction

FTIR spectra of black cardamom extract have been recorded before and after bio reduction of HAuCl_4_ to reveal and understand the formation mechanism of nanoparticles as shown in Fig. 5.

This spectrum provides the information of changes in functional groups of chemicals, basically found in black cardamom extract which was further utilized to deduce a plausible mechanism for the bio reduction of gold nanoparticles. Sample for FTIR of black cardamom extract after the reduction of gold nanoparticles has been obtained by the centrifugation of reaction mixture at 15000 rpm for nearly 15 min. In this process, the particles get settled at the bottom and supernatant have been collected for further analysis. One milliliter of black cardamom extract before and after the bio reduction of HAuCl_4_ has further been employed for recording the FTIR spectra. FTIR spectra of black cardamom extract before reduction clearly show a large number of peaks from 4000 to 3200 cm^−1^_._ Around 3200 cm^−1^, there is a broad peak which attributes –OH.

However, this radical disappears in the spectra recorded after the bio reduction, which indicates the consumption of –OH in the reduction of HAuCl_4_. Rest other bands up to 1800 cm^−1^ appear in both the spectra, i.e., spectra obtained before and after the reduction, are common. Below 1800 cm^−1^, a new band at 1770 cm^−1^ appears after the reduction. This is the fingerprint signature of –C=O functional group in stretching mode. After 1770 to 500 cm^−1^, positions of bonds are again almost similar as those before reduction, with varying intensity.

These two changes in the spectra of before and after the bio reduction are of significant interest. They provide fruitful information regarding the formation mechanism of gold nanoparticles. This analysis can be summarized as follows

- After the formation of gold nanoparticles, –OH (visible at 3200 cm^−1^) group disappears.
–C=O group (1770 cm^−1^) appears after the formation of gold nanoparticles.
- Rest other groups like (R–CH) are common in both the spectra.

Based on the above FTIR results, attempts have been made to propose a viable growth mechanism of gold nanoparticles, as discussed in the next section.

### Reduction Mechanism of Gold Nanoparticles

To understand the change in –OH group that whether this change is due to the chemical-containing –OH group, which is present in the aqueous black cardamom extract medium or due to the aqueous medium itself. In order to check this, the experiment has been repeated in organic solvent with no –OH group like hexane (though HAuCl_4_ has low solubility in organic solvent). The results have not shown any indication of formation of gold nanoparticles which leads to conclude that the presence of water molecule is an essential part for the reduction of energy.

**Fig. 6:**
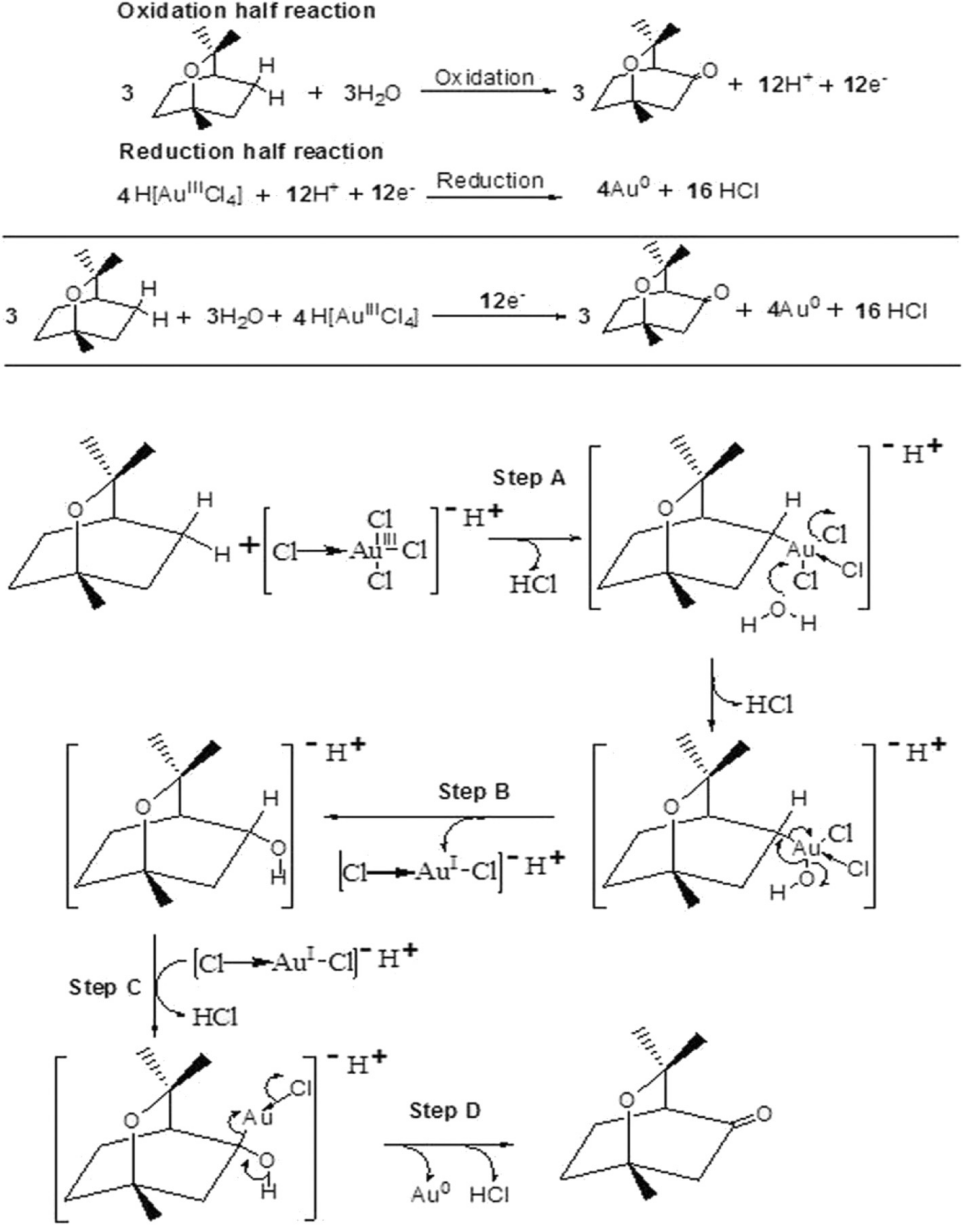
Full redox reaction and plausible mechanism for the synthesis of gold nanoparticle by oxidation of 1,8-cineole

In this connection, feasible schematic diagram for the formation of gold nanoparticles has been proposed, which is shown in Fig. 6. HAuCl_4_ is an ionic compound containing H^+^ and AuCl_4_^−^ ions, where Au has +3 oxidation state in AuCl_4_^−1^ and three Cl atoms are covalently bonded while rest one Cl atom is coordinately bonded. Since all atoms are identical, therefore, they keep on interchanging from coordinate to covalent bonding and vice versa.

### Step A

When the reaction starts, coordinately bonded Cl reacts with α-H of 1,8-cineole followed by the removal of HCl and forming a bond between Cl and Au^3+^. Here, Au is again in +3 oxidation state, therefore, one Cl atom will get coordinately bonded with Au.

### Step B

Since water was used as medium in this experiment, in which oxygen molecule have two pairs of electron, therefore, one of these two lone pairs attacks on Au to form Au-O bond. This bond formation leads to the removal of HCl where Cl^−^ and H^+^ atoms came from Au and water, respectively.

### Step C

Now, the lone pair of –OH attacks on C, therefore, carbon gives its bond pair to Au to maintain its oxidation number zero. Since oxygen has given its lone pair to carbon, it will acquire a positive charge, which will be neutralized by elimination of H^+^ after the removal of Cl^−^ from AuCl to form HCl. In this step, Au^3+^ gets reduce to Au^+^

### Step D

In this step, C–H Bond pair migrates to form C=O and H^+^ gets removed by forming HCl. Further, the bond pair between O and Au moves to Au^+^ which leads to the reduction of Au+ to Au

The above described mechanism can be summarized through schematic chemical diagrams shown in Fig. 6.

$$ 4\ {\mathrm{H}}^{+}{\left[\mathrm{A}{\mathrm{u}}^{3+}\mathrm{C}{\mathrm{l}}_4\right]}^{\hbox{-} } + 12\ {\mathrm{H}}^{+}+12{\mathrm{e}}^{\hbox{-}}\to 4\ \mathrm{A}{\mathrm{u}}^0 + 16\mathrm{H}\mathrm{C}\mathrm{l} $$

### Effect of pH on the Synthesis of Gold Nanoparticles

For investigating the role of pH on the synthesis of gold nanoparticle, a separate set of experiments have been performed with variation in pH of HAuCl_4_ ranging from 3 to 11 by addition of different amount of 1 M NaOH solution. Five test tubes, each containing 10 mL of 0.001 m HAuCl_4_, have been taken, and pH was maintained carefully from 3 to 11 with step size of 2. Amount of NaOH, which is required for maintaining the pH as desired and some other useful UV-visible spectroscopic results are presented in Table 1.

**Table 1:** Variation in absorbance maxima with change in pH synthesis of gold nanoparticle by oxidation of 1,8-cineole

Sample Name	Amount of NaOH Added (μL)	pH of Solution Before Reduction	pH of Solution After Reduction	Absorbance Maxima (nm)
S1	12	3.03	4.24	572
S2	37	5.09	5.04	560
S3	55	7.04	6.01	544
S4	70	9.10	6.98	548
S5	85	11.08	7.67	562

### UV-Visible Spectroscopic Study

**Fig. 7:**
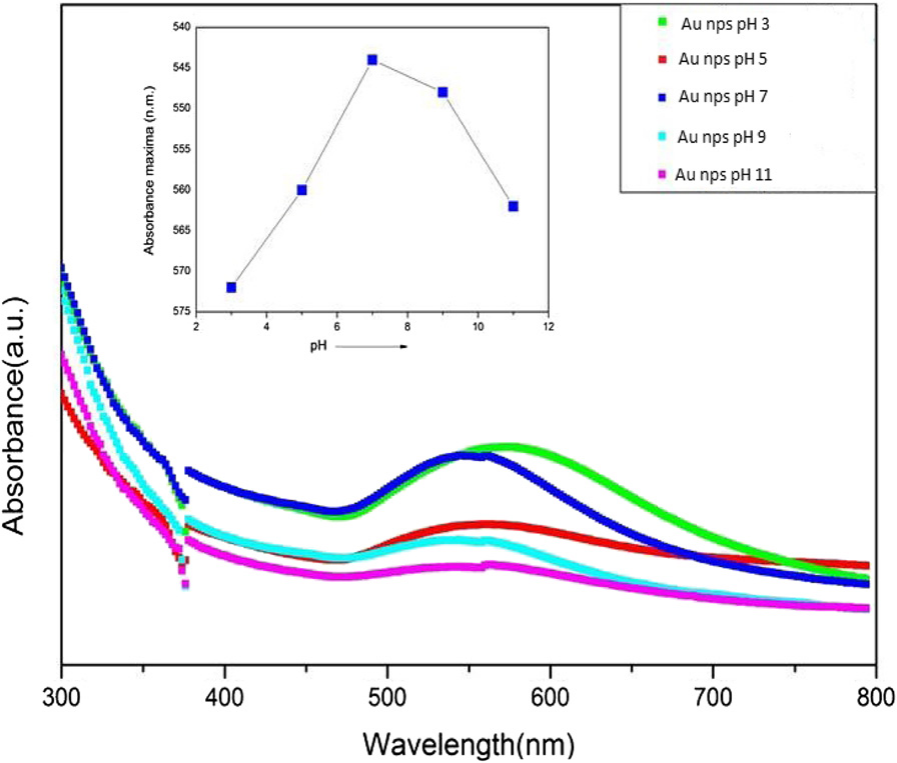
UV-vis spectra of Au nanoparticles synthesized at different pH

These synthesized materials immediately undergo for UV-visible spectroscopic study for further investigation. One milliliter of reaction mixture diluted with 3 mL double distilled water has been employed for recording the spectrum. These recorded spectra have been shown in Fig. 7. The observed absorption maxima for gold nanoparticles are at 572, 560, 544, 548, and 562 nm for the solution having pH values 3, 5, 7, 9, and 11, respectively. This analysis explicitly depicts that there is a gradual shift in absorption maxima towards lower wavelength side with an increasing pH from 3 to 7. This shift in absorption maxima gives a clear indication that the sizes of the particles decrease when pH changed from highly acidic to neutral i.e., from 3 to 7. If the pH is further increased from 7 to 11, the observed shift in absorption maxima is towards red region, i.e., towards higher wavelength region. This shift suggests that size of the particles increased when pH is increased from 7 to 11. Therefore, in a nut shell, it can be said that in the spectroscopic study of synthesize gold nanoparticles as the particles of smaller size, they can be synthesized when the pH of the solution is at or around 7.

### TEM Analysis

**Fig. 8:**
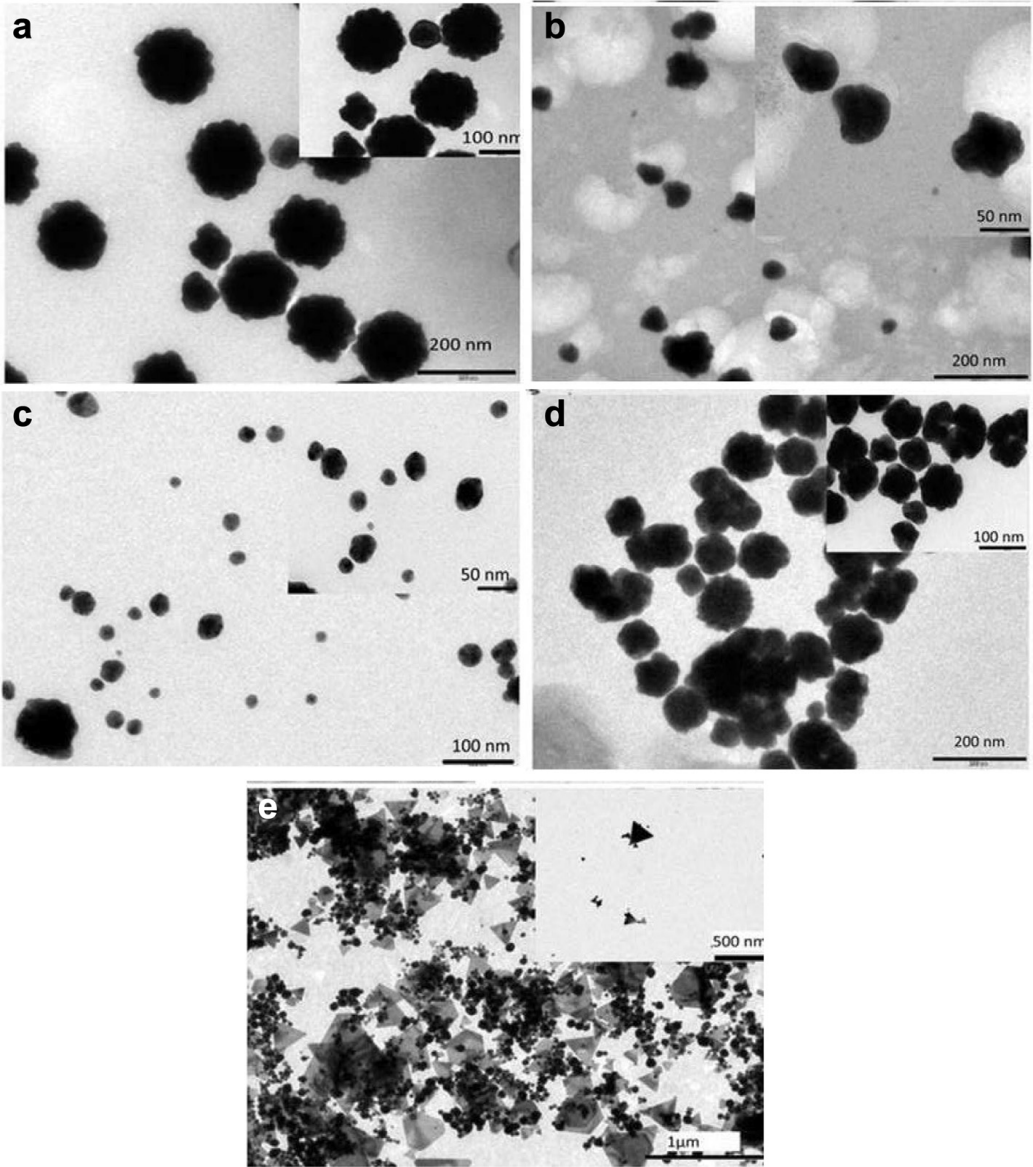
TEM micrograph of gold nanoparticles synthesized at different pH of **a** pH 3, **b** pH 5, **c** pH 7, **d** pH 9, and **e** pH 11

Transmission electron microscopic studies have further been carried out to analyze structural characteristics and support UV-visible spectroscopic analysis. Figure 8a shows TEM micrograph of gold nanoparticles synthesized at pH 3. This micrograph shows a spherical flower shaped morphology with average particle size of nearly 90–100 mm and this morphology is more clearly visible in magnified image, as shown in the inset of Fig. 8a. A close observation shows that the particles have ripples on the surface of the particles which makes a flower-like structure of gold nanoparticle. In Fig. 8b, the morphology of particles is of irregular shape but the size of the particles has decreased. These nanoparticles have been synthesized when the pH of the solution was maintained at 5. The inset picture shows clearly that the particles have no definite shape. Particles under pH 7 condition are nearly spherical in shape with an average particle size of 20–40 nm. After observing Fig. 8d, e carefully, it can be concluded that there is a sharp change in shape and size of the particles as well. In Fig. 8d, particles are nearly irregular in shape (pH 9) while in the next Fig. 8e, mainly two types of particles are formed, one almost spherical and others are triangular in shape.

### XRD Analysis

**Fig. 9:**
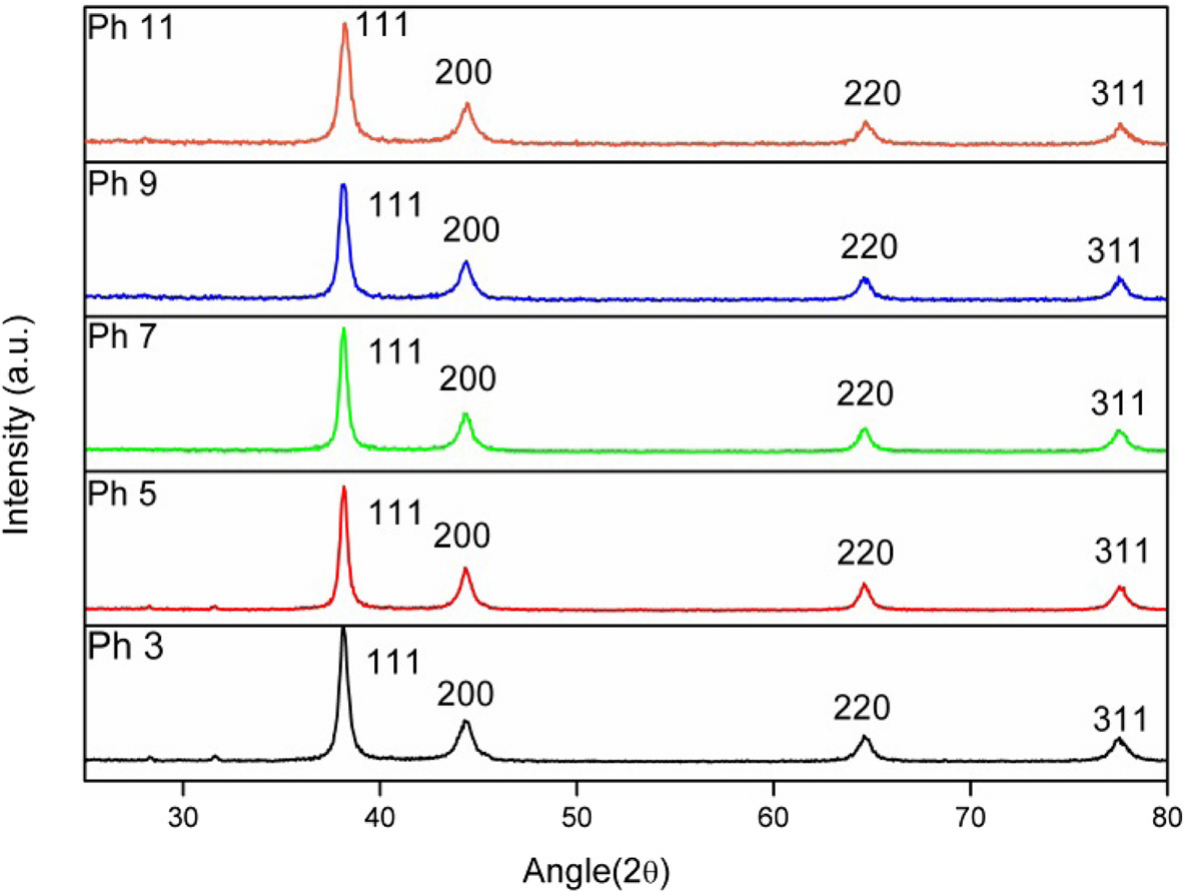
XRD patterns of gold nanoparticles synthesized under different pH conditions

X-Ray diffraction analysis was carried out in order to investigate the crystallinity and change in *d* value of gold nanoparticles. Figure 9 shows the representative X-ray diffractograph of gold nanoparticles synthesized under different pH conditions. The peaks, which are labeled as 111, 200, 220, and 311 indicate the formation of crystalline gold nanoparticles. These patterns also show no *d* value change due to the variation in pH, as it is clear that all similar planes in XRD peaks corresponds to the same 2θ value.

## Conclusions

In conclusion, a simple, one-step, and fast green route for the synthesis of gold nanoparticles of different shapes and sizes has been demonstrated. It has been found that these physical parameters (shape and size) can be tuned easily by varying the ratio of the HAuCl_4_ to black cardamom extract. The UV-visible spectroscopic study provides some clue about the evolutions of nanoparticles with time and variation in sizes at the same time. These observations can further be confirmed by histogram plot of particle size distribution obtained from TEM analysis. These analyses lead to the conclusion that the size of particles increased when the concentration of black cardamom extract is decreased. The XRD analysis explicitly shows the formation of gold nanoparticles as the diffraction peaks match well with the standard value of gold lattice structure. Reduction of HAuCl_4_ takes place because of 1,8-cineole. A plausible mechanism of reduction has been put forward, based on analysis of FTIR spectra of black cardamom extract before and after the bio reduction of HAuCl_4._
